# Professor Tafu Yu: an eminent agricultural scientist and outstanding educator of our nation

**DOI:** 10.1007/s13238-017-0494-3

**Published:** 2018-02-08

**Authors:** Shuang Zhao, Tiantian Xu, Hexiang Wang

**Affiliations:** 1 Institute of Plant and Environment Protection, Beijing Academy of Agriculture and Forestry Sciences, Beijing Engineering Research Center for Edible Mushroom, Beijing, 100097 China; 2 State Key Laboratory of Agrobiotechnology and College of Biological Sciences, China Agricultural University, Beijing, 100193 China

**Figure 1:**
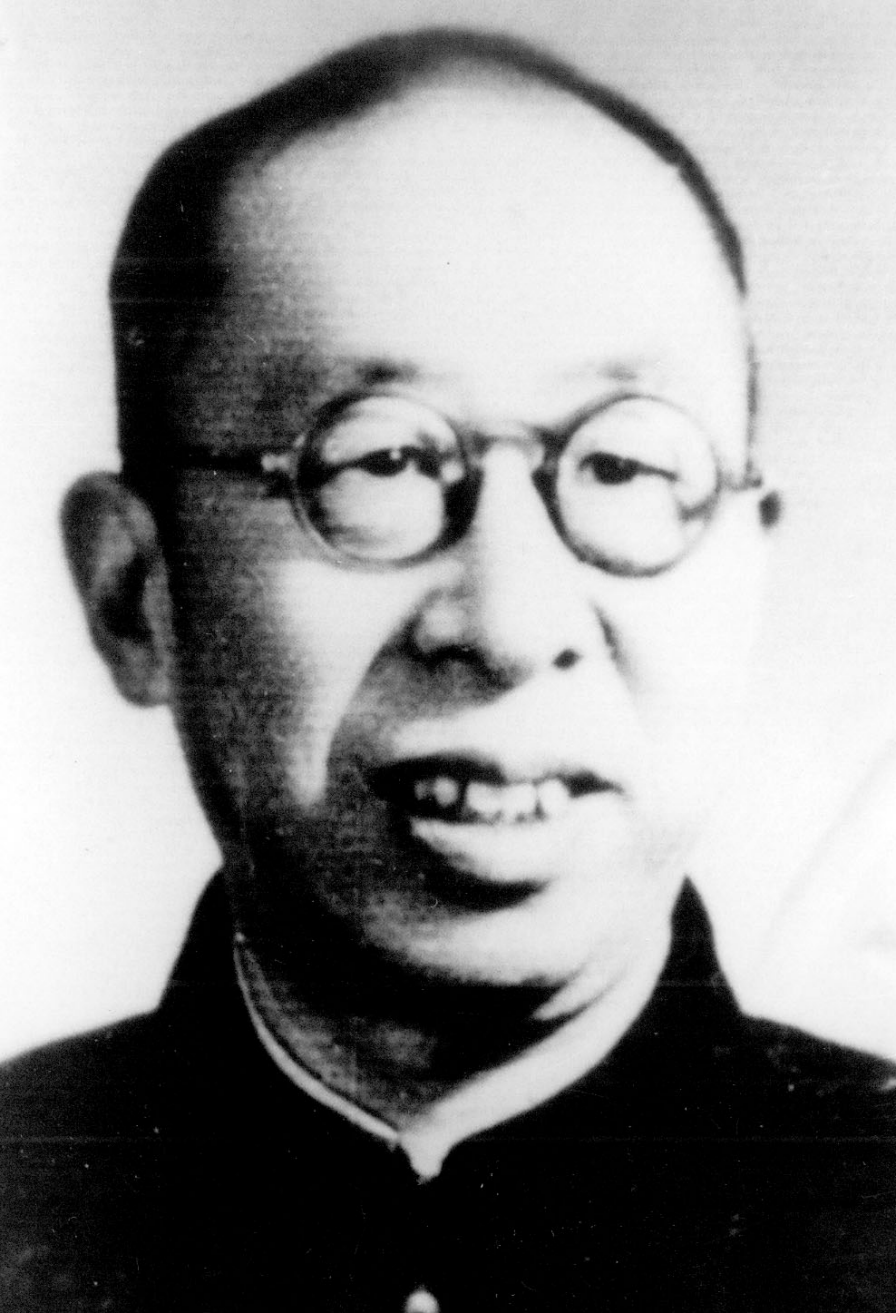
Prof. Tafu Yu (1901–1993)

Prof. Tafu Yu (1901–1993) (Fig. 1) is a pioneering scientist of phytopathology and agricultural microbiology in China. Like most of the scientists of his generation, Prof. Yu gave up the attractive conditions abroad and went back to China with the determination to rescue the nation with his knowledge. He has devoted his entire life to the development of phytopathology in China since 1924. Prof. Yu is also a distinguished educator who has instructed and inspired several generations of talent students for the country and has made outstanding contributions to the development of the agricultural science and education in China.

Prof. Yu was born in 1901 in a well-educated family in Nanjing, Jiangsu Province. At that time, China was suffering from domestic strife and foreign invasion, with weakness and poverty being the major issues. Prof. Yu’s parents hoped that their children could study different disciplines so that they can use their knowledge later on to rescue the country in different ways, such as science, education and industry. Prof. Yu gave up his original major in physics and chemistry and decided to study agriculture in order to solve the problems of poverty, backward agriculture and severe plant diseases for China. In 1924, he obtained his bachelor’s degree from the College of Agriculture in Nanjing Jinling University. During that time, he also worked as a teaching assistant. In 1928, Prof. Yu continued his study in Iowa State University in the United States and received his doctoral degree and a Golden-Key award in 1932. Then he became a member of the American Phytopathological Society and Sigma-Xi. He also received a “Phi Beta Kappa” award of the United States.

**Figure 2:**
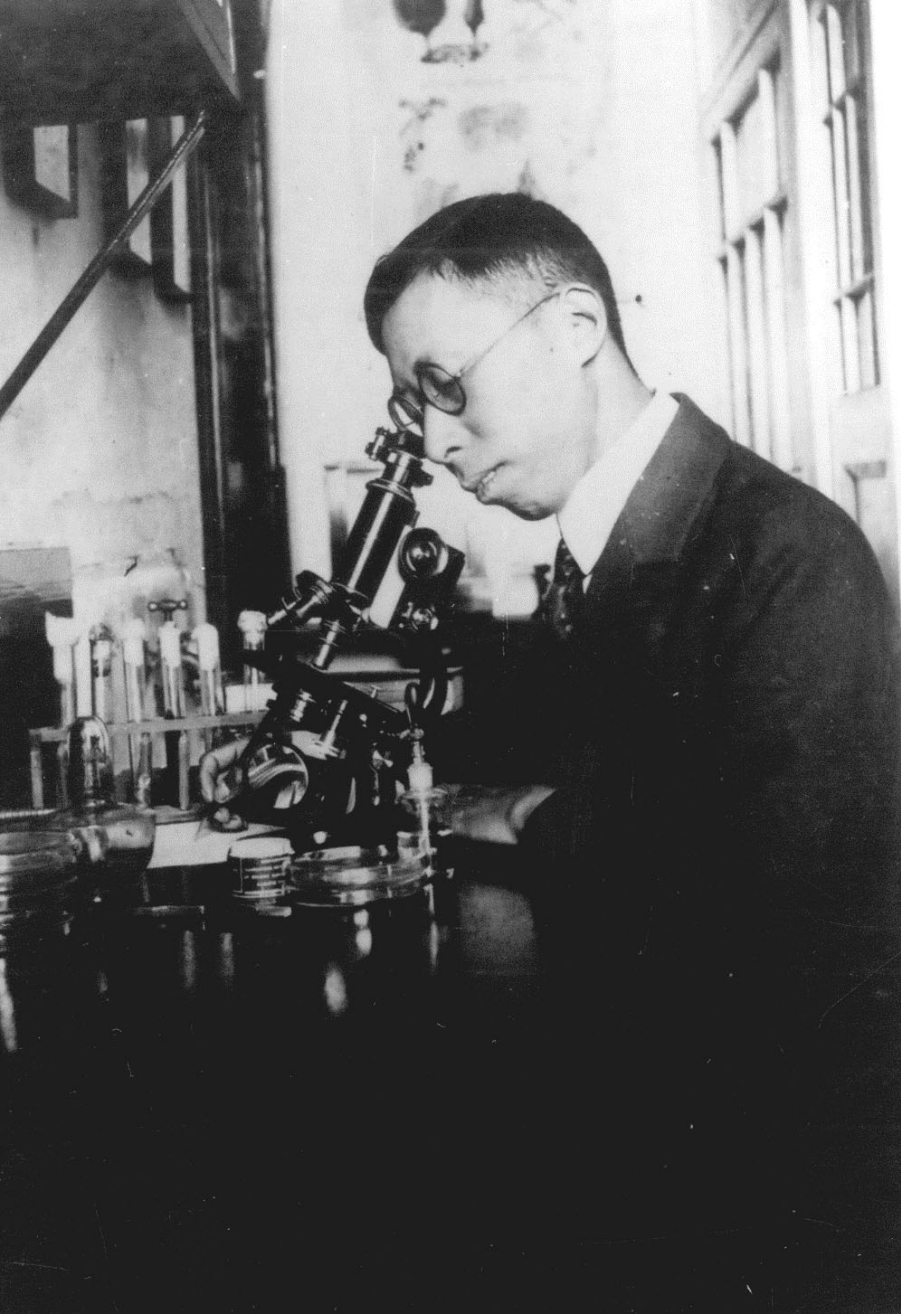
Tafu Yu worked in laboratory at Nanjing Jinling University

Prof. Yu returned back to China after graduation in 1932 and served as a professor at Nanjing Jinling University (Fig. 2), and later on as a professor at the Agricultural Research Institute of Tsinghua University. He was then appointed as director and professor of the College of Agriculture in Peking University, prior to taking up the positions of academician and senator of the Central Research Institute. Following the founding of People’s Republic of China, he started his career as an educator at Beijing Agricultural University as the President. In 1955, he was elected as one of the first generation of academicians of the Chinese Academy of Sciences and one year later, he was elected as the Communications Academician of the Soviet Academy of Agricultural Sciences. Prof. Yu was a member of the second and third CPPCC national committee and a member of the fourth, fifth and sixth standing committee (Qing, 2007). He also served successively as vice president of Chinese Association of Agricultural Science Societies, and chairman of China Society of Plant Protection and chairman of Chinese Society for Plant Pathology.

Prof. Yu was a pioneer of modern plant pathology and also the authority of plant bacteriology, plant virology, plant disease resistance breeding, disease physiology and other areas in China. He studied crops such as cereal crops, beans, fruit trees and vegetables, as well as crop pathogens including fungi, bacteria and viruses. He adopted the prevention and control strategies, including seed treatment, chemical prevention and control, cultivating disease-resistant varieties etc. Prof. Yu discovered that *Urocystis tritici* exhibited the characteristic of physiological differentiation and initiated the researches on physiological breedings in China. Prof. Yu published more than 110 research articles in international and domestic academic journals, 10 monographs and 8 translated articles. In the 1920s and 1930s, Prof. Yu investigated the breeding of disease-resistant cereal crops and seed disinfection methods, clarified the infection patterns of barley smut and stripe disease, bred crop varieties such as smut-resistant wheat, blight-resistant soybean, and blast-resistant rice. He also launched a study on the physiological strains of the smut fungus.

In 1940s, Prof. Yu was invited to teach in Tsinghua Agricultural Research Institute in Southwest Associated University in Kunming. At the time during the War against Japanese aggression, he conducted scientific researches under extremely difficult conditions. He discovered that *Urocystis tritici* displayed the characteristics of physiological differentiation and performed investigations on diseases that occurred during fruit storage. Concurrently, he also carried out a comprehensive study on the diseases that affected the millet and broad bean. Prof. Yu published numerous scientific papers which represent a classic literature of many critical plant diseases in China.

**Figure 3:**
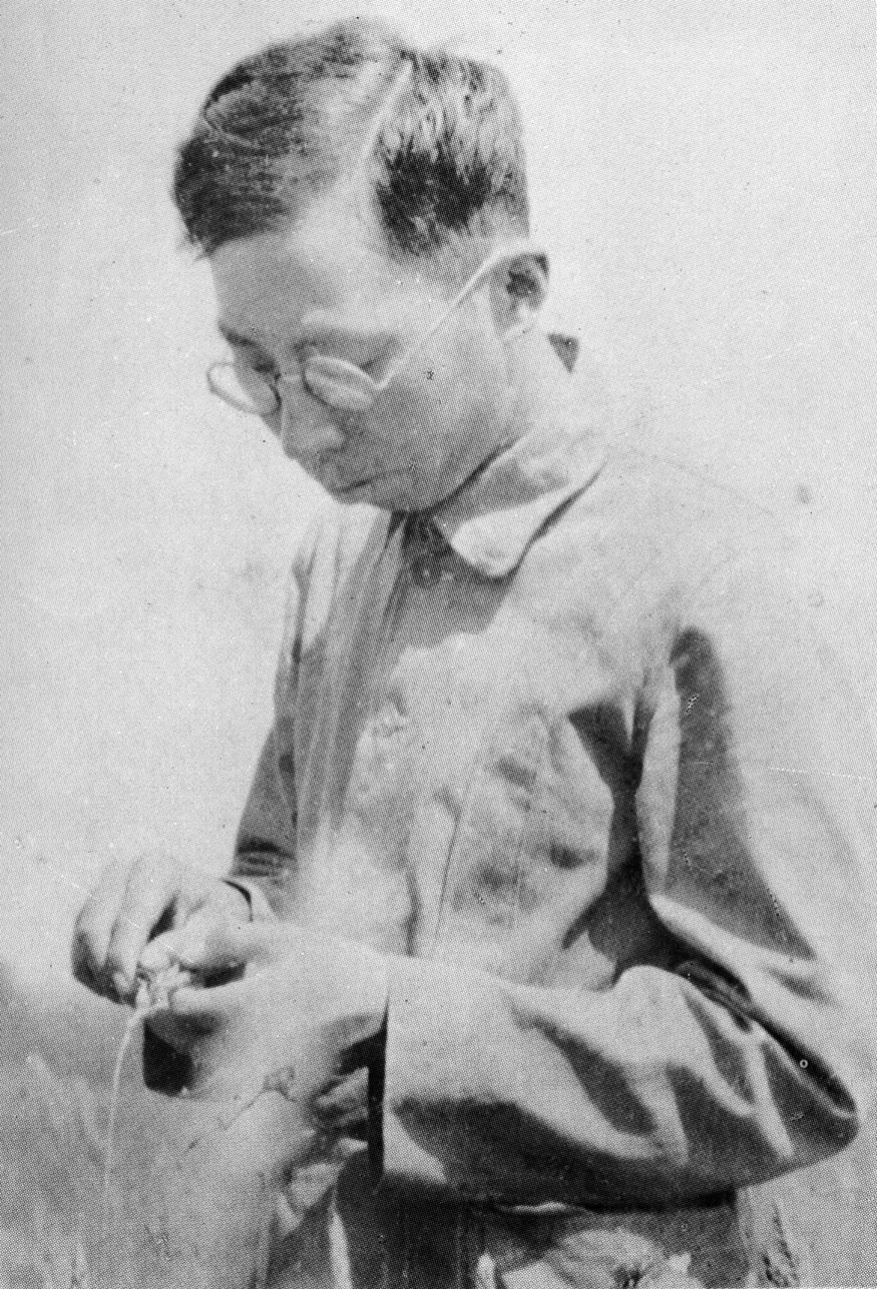
Tafu Yu observed the plant diseases in the field

After the founding of People’s Republic of China, Prof. Yu had more freedom for his scientific researches. At that time, large area of farmland in the country was damaged by plant diseases, such as apple valsa canker disease, potato late blight disease, millet red leaf disease and citrus scab disease (Yu, 1951). Appointed by the Ministry of Agriculture, Prof. Yu led a research team to investigate the affected farmland (Fig. 3), to prevent the spreading of the plant diseases, they try their best to conduct in-depth researches, execute effective prevention and control methods (Yu, 1953). He trained a number of plant quarantine personnel for the prevention and control of plant diseases, which laid the foundation of the plant disease quarantine work in China. In 1955, he was honored as a member of the first Chinese Academy of Science Committee and his work was highly praised by the Party Central Committee and the State Council. In 1958, Prof. Yu and his colleagues carried out researches on gibberellins in Beijing Agricultural University, where they developed the *Gibberella zeae* strains and filled in the gaps in gibberellin research and production in China (Rong et al., 2017). During the 1960s, Prof. Yu mainly focused on the basic researches and studied fungal heteronuclear genetics, classification and identification of Fusarium species as well as selected breeding of high-yield *Gibberella zeae* strains (Yu, 1977). His studies revealed that *Gibberella zeae* formed heterokaryons in nature from three different karyotypes, and proved that strains with different heterokaryons varied in gibberellin production and parasitism. His findings effectively demonstrated whether the heteronuclear phenomenon prevailed in nature, a long-term controversial question. It not only confirmed that fungal heterokaryon was implicated in mutations of pathogenic fungi, but also improved the targeting of breeding for disease-resistance. His achievements facilitated the upgrading of the methods for prevention and treatment of plant diseases from passive therapy to active prevention, which had a significant long-term impact on agricultural production. This achievement attained an advanced scientific level and was awarded the first prize of Science and Technology Achievements from Ministry of Agriculture, Animal Husbandry and Fisheries in 1980. Prof. Yu’s researches implemented an intimate association between solving agricultural production issues and promoting the development of agricultural producing system. Prof. Yu spent his entire lifetime to tackle the diseases encountered in agricultural production in China. He has made spectacular accomplishments for China’s agricultural production practice conditions, which profoundly enhanced the development of agriculture in China.

Besides devoting himself to agricultural researches, Prof. Yu was also dedicated to agricultural education. He was truly a prestigious and influential agricultural educator of China. After the victory of the Anti-Japanese War, Prof. Yu was appointed to rebuild the College of Agriculture in Peking University. He invited a large number of distinguished scholars from abroad to give lessons in the College of Agriculture, established a complete discipline framework and teaching management system and advocated a democratic and open minded education system. Prof. Yu’s patriotism was highly respected. He opposed the Kuomintang (KMT) reactionaries who suppressed students’ patriotic and democratic movement, and supported the patriotic activities of teachers and students. He rejected the invitation of Nanjing Kuomintang (KMT) government, remained unwaveringly in his position and protected the campus and properties of the university. Meanwhile, he accepted the invitation of the communist party, stayed with the teachers and students in the university and then transported important equipment to the liberated areas and resumed scientific researches there.

**Figure 4:**
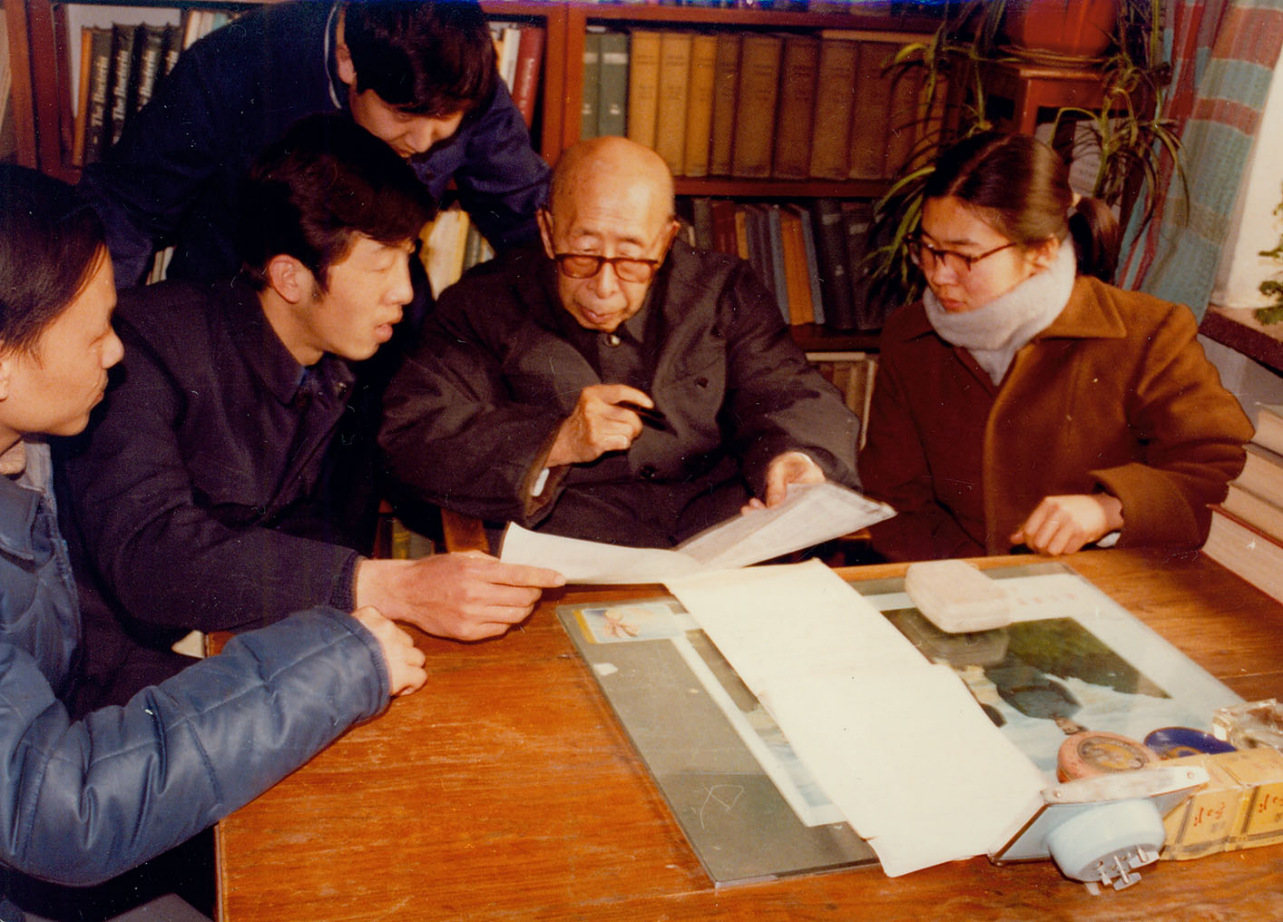
Prof. Yu supervised the students to do research

His eminent deeds were recorded in the documentary “Love of The Republic “. In the 1980s, at the age of 80, Prof. Yu took the position of the President at Beijing Agricultural University again. He proposed the concept of combining “agriculture” and “engineering” for running the College of Agriculture and promoted the development of the Agricultural University toward a university of agriculture, science and technology. Being a teacher for over 70 years, Prof. Yu educated several generations of talented students (Fig. 4). Many of his students followed his footsteps and continued to make tremendous contributions in areas of education, science and management. Some of them are well-known, such as Zhongda Fang, Chuanguang Li and the academicians Wenxin Chen, Jilun Li (Rong et al., 2017), Weifan Qiu and Shimai Zeng.

Prof. Yu, a highly respected scientist, has dedicated his entire life to the development of agriculture in China. He laid the foundation for plant pathology and agricultural microbiology in China. There is a famous Chinese proverb: It takes ten years to grow trees, but a hundred years to rear people. Prof. Yu not only imparted knowledge, but also educated his students how to behave. His spirit and exemplary roles influenced generations of young researchers and his prominent contributions to the development of China’s agriculture will never be forgotten.
